# When goodbye comes too soon: How to wrap up science projects quickly

**DOI:** 10.1371/journal.pbio.3003455

**Published:** 2025-10-21

**Authors:** Megan Hastings Hagenauer, Stacey J. Winham, Alexandra L. J. Freeman, Paul W. Sternberg, Benedict J. Kolber

**Affiliations:** 1 Michigan Neuroscience Institute (MNI), University of Michigan, Ann Arbor, Michigan, United States of America; 2 Division of Computational Biology, Mayo Clinic Rochester, Rochester, Minnesota, United States of America; 3 Octopus Publishing CIC, Abingdon, United Kingdom; 4 Division of Biology and Biological Engineering, Caltech, Pasadena, California, United States of America; 5 Department of Neuroscience, University of Texas at Dallas, Richardson, Texas, United States of America

## Abstract

Science projects are designed and funded on the scale of years, so what happens when researchers need to finish prematurely? This Community Page discusses solutions for quickly documenting partially finished projects, and for publishing ‘as you go’ in an uncertain environment.

## Teaser

Science projects are designed and funded to last several years, but sometimes end prematurely, leading to lost data and effort. We discuss inexpensive solutions for quickly documenting partially finished projects and for publishing “as you go” in an uncertain environment.

Science is an assiduous endeavor. Projects are often designed and funded on the scale of years or decades. Even a completed project, fully written up, may spend many months undergoing peer review and revision prior to publication. And painfully, until the work is published, the public impact of complex scientific efforts is minimal.

In such a slow system, what can we salvage when multi-year projects need to end prematurely? This issue has come to the forefront recently due to the rapid reduction of US federal science funding and infrastructure [1]. However, even under typical circumstances, many well-designed projects are never completed due to a lack of time or funding, and research can be disrupted as a result of career changes, visa issues, or personal circumstances. Transferring responsibility for a project to a collaborator may cause it to stall, or may simply be impossible, especially if the entire project is defunded or the laboratory closes.

Premature project terminations threaten to waste existing public and private investments [2], and forfeit hard-earned knowledge. There is, therefore, an imperative to establish a scientific record of current progress efficiently and quickly. In this Community Page, we discuss existing venues and resources for rapidly documenting projects that have not reached their natural conclusion, as well as publishing “as you go” to prevent future problems in an uncertain environment. Importantly, these resources allow documentation in a manner that provides credit for individual efforts and accomplishments, enabling future researchers to build on unfinished work.

## Where to publish half-finished projects

Within most scientific disciplines, it is traditional to wait to publish a study until the results provide a strong answer to a research question. However, there is active debate as to whether this is the ideal workflow. Under this model, an estimated 40% of data go unpublished [3], with a strong skew against publishing negative results [4]; a source of bias in the scientific literature referred to as “The File Drawer Problem.” Emphasizing a publishable “story” may also encourage questionable research practices, such as the flexible “dredging” of data to achieve statistically significant results (P-hacking), adapting hypotheses to match results (HARK-ing), and the selective reporting of findings (cherry picking) [5].

In response, there has been a growing movement for “modular publishing,” or sharing smaller component parts of a study as they are produced [6]. Within this model, components can be publicly released, either in specialized repositories as non-peer-reviewed interim products, or published separately in dedicated peer-reviewed journals (Table 1). There are also formats that encourage such staging, allowing components to be formally linked or released together, sometimes with opportunity for postpublication peer review and revision (e.g., Open Science Framework (OSF), Octopus.ac, ResearchEquals, and Research Ideas and Outcomes). Individual complete experiments can also be documented in short formats instead of being grouped together to build a larger “story.” In general, publication in specialized repositories is free and rapid (on the scale of days), whereas traditional journal publication is often accompanied by article processing charges and is much slower due to the editorial and peer review process, which may involve reviewer-requested experiments, or rejection and resubmission.

**Table 1:** Example venues for releasing or publishing products produced at each stage of the research process. In this table, we emphasize existing options that offer unique, persistent digital online identifiers (DOI) and indexing to facilitate product discovery and access. A version of this table can also be found at https://doi.org/10.6084/m9.figshare.29356853.v3 with updated hyperlinks and relevant additions as they become available.

Stage of project	Non-peer-reviewed products	Peer-reviewed products
**Hypothesis, research problem, or proposal**	***Compatible preprint servers:*** Octopus.ac, Preprints.org, ResearchEquals, ResearchSquare (for others, see ASAPbio)	***Research proposal publication:*** Research Ideas and Outcomes ***Review article:*** Most scientific journals (to search: Jane)
**Vetted protocol**	***Protocol repositories:*** OSF, Protocols.io	***Protocol publication:*** BMC Methods, F1000Research, GigaByte, JoVE, Lab Animal, Nature Protocols, PLOS One
**Methods paper or technical note**	***Compatible preprint servers:*** Octopus.ac, Preprints.org, ResearchEquals, ResearchSquare (for others, see ASAPbio)	***Methods papers or technical notes:*** Biological Procedures Online, Cell Reports Methods, Methods, MethodsX, Nature Methods, PLOS Biology, PLOS Computational Biology (for others, see Jane and Wikipedia)
**Data release or data note**	***Data release repositories:*** Dryad, figshare, Mendeley Data, Zenodo (for disciplinary repositories: DataCite, FAIRsharing or re3data) ***Compatible preprint servers:*** Octopus.ac, Preprints.org, ResearchEquals, ResearchSquare (for others, see ASAPbio)	***Data release paper or data note:*** BMC Research Notes, Data, Data in Brief, F1000Research, GigaByte, Open Research Europe, Scientific Data (for others, see this List of Data Journals)
**Algorithm, tool, or software**	***Code or software release repository:*** Code Ocean, GitHub, Zenodo ***Compatible preprint servers:*** Octopus.ac, Preprints.org, ResearchEquals, ResearchSquare (for others, see ASAPbio)	***Tool or software note:*** F1000Research, Journal of Open Research Software, Journal of Open Source Software, PLOS Computational Biology, SoftwareX (for others, see The Software Sustainability Institute)
**Partial analysis, preliminary analysis, or small/limited study**	***Poster servers:*** F1000Research, PeerRef, ResearchEquals, ScienceOpen ***Research lecture notes or conference proceedings:*** arXiv, ResearchEquals ***Generalized repositories:*** figshare, Zenodo ***Short result preprint:*** Octopus.ac, ResearchEquals (for others, see ASAPbio)	***Micropublication, brief report, short report, brief communication, or short communication:*** BMC Research Notes, F1000Research, Lab Animal, microPublication.org, PLOS Biology, Research Ideas and Outcomes, many field specific journals (see Jane)
**Thesis or dissertation (no time to reformat)**	***Compatible preprint servers:*** arXiv, OSF Thesis Commons, ResearchEquals	***Peer-reviewed preprints:*** Peer Community in, PREreview, ResearchHub, ScienceOpen ***Post-publication review:*** Research Ideas and Outcomes
**Full manuscript (no time for lengthy peer review)**	***All preprint servers:*** arXiv, bioRxiv, Preprints.org (for others, see ASAPbio)	***Peer-reviewed preprints:*** eLife, Peer Community in, PREreview, ResearchHub, Review Commons, ScienceOpen ***Post-publication review:*** Copernicus Publications, F1000Research, Open Research Europe, Sci

Online tools are available to help match publishing solutions to the needs of the researcher. The online tool Fiddle can match scientists with peer-reviewed and non-peer-reviewed options, taking into account individual financing and indexing constraints. The online tools Jane and Journal Finder recommend journals using the abstract text and/or title, and Transpose provides an easy way to search for journals with particular policies that can expedite publishing [7], including peer review transfers between journals (although this database is no longer actively maintained). ASAPbio also hosts a searchable database of preprint servers, and re3data, FAIRsharing, and DataCite have extensive guidance regarding field-specific data repositories.

## Is there a down-side to releasing research products piecemeal?

There are reasons not to publicly release partially finished projects. Some research may be incompatible with these formats owing to ethical constraints on the public release of human subjects data or sensitive animal data. There may also be concerns that publication of research components may hinder later attempts at traditional publication. To this end, most major journals have begun to welcome, or even encourage, the separate release of study components [8], as this relieves the final publication of the burden of providing the full study documentation necessary for research reproducibility. The vast majority of journals also welcome manuscripts that have been released as preprints [7], with some even accepting direct transfer of preprints (and sometimes their reviews) to journal submission systems. In the meantime, these modular research outputs can be reported on CVs and grant applications to illustrate productivity, skills, and dedication to open science practices.

Another concern is that “unfinished” projects may lack replication or validation work. The publication of such data without acknowledging its exploratory nature may lead to wasted future effort. However, if limitations are properly acknowledged, contributing exploratory work to the scientific record can benefit future meta-analyses and encourage replication attempts.

## Wrapping things up quickly

Although the options described in Table 1 for modular publishing or preprinting are faster than traditional scientific publications, preparing these products still requires effort and time. All formats require gathering coauthor approval and conflict of interest disclosures, which can take several weeks, and peer-reviewed products will still require both editorial and peer review, and often revision and re-review (for individual journal time estimates, see SciRev.org). Furthermore, the products should be carefully prepared and cleanly formatted, as they will be joining the permanent public record and their utility will depend on the quality of their description.

To this end, scientists who still have access to resources may consider offloading some work onto a professional science writer, paper reformatting service (e.g., Editage) or scientific editing service (e.g., those offered by SpringerNature or Elsevier). If so, it is important to select a writer or service with suitable training and qualifications, and to properly define, oversee, and acknowledge their involvement, as ghost authorship is ethically prohibited in most contexts. Moreover, substantial contributions to interpretation may require consideration for full authorship [9].

Artificial intelligence (AI) tools can also help with browsing literature, summarizing rough notes, digitizing protocols, organizing code or data for release, improving data visualizations, improving grammar, clarity, tone, or conciseness of text, and helping reformat a paper to fit journal requirements [10]. Researchers need to proceed with AI tools carefully. Some tools will store and reuse inputted data, which is incompatible with sensitive data (e.g., clinical data). It is also important to double-check AI output for inaccuracy, inappropriate use or misinterpretation of source material and hallucinations [11]. Moreover, the paper must still center on the authors’ own work and ideas, and all AI usage must be carefully documented and transparently disclosed, following journal requirements [9].

## What about money?

Many of the publishing outlets in Table 1 are free, but some charge fees. Ideally, costs such as article processing charges would be charged to grants, as it is in the funders’ best interest for all research products to be publicly shared. If funding is unavailable, it is worth contacting your institution’s library or grants office, if available, to determine if any journals or publishers have an open access agreement or fee waiver for your institution or research society (e.g., the Big 10 Academic Alliance). Many journals also offer fee waivers or reduced charges for researchers from low- and middle-income countries (e.g., Research4Life), or in exchange for reviewing papers.

What about the cost of preparing the publications and research products? At this point, there are few sources of support for scientists interested in cleaning out their file drawers. The American Association of University Women offers grants to finish dissertation writing or prepare manuscripts for publication, and publication grants are occasionally available at the institution level. Offering grants to defunded or transitioning researchers for rapid research product release and manuscript preparation would be a high-impact investment for institutions and research foundations, yielding high returns for minimal upfront costs.

## Conclusions

Due to a variety of circumstances, including sudden policy shifts and common personal challenges, many science projects will never be finished, despite years of invested resources and effort. By carefully and strategically documenting scientific work achieved, components of unfinished projects can be salvaged and preserved to benefit future researchers. Moreover, by thoughtfully considering options for modular publishing before they are needed, scientists can be better prepared for unexpected changes to their trajectory and careers.

## Abbreviations

AI: artificial intelligence
DOI: digital online identifiers
OSF: open science framework

## References

[1] Tollefson J, Garisto D, Ledford H. Will US science survive Trump 2.0? Nature. 2025;641(8061):26–30. doi: 10.1038/d41586-025-01295-6 40301614

[2] Larson RC, Ghaffarzadegan N, Diaz MG. Magnified effects of changes in NIH research funding levels. Service Sci. 2012;4(4):382–95. doi: 10.1287/serv.1120.0030 PMC3908662 24489978

[3] Bowers EC, Stephenson J, Furlong M, Ramos KS. Scope and financial impact of unpublished data and unused samples among U.S. academic and government researchers. iScience. 2023;26(7):107166. doi: 10.1016/j.isci.2023.107166 37485349 PMC10359936

[4] Fanelli D. Negative results are disappearing from most disciplines and countries. Scientometrics. 2011;90(3):891–904. doi: 10.1007/s11192-011-0494-7

[5] Andrade C. HARKing, cherry-picking, p-hacking, fishing expeditions, and data dredging and mining as questionable research practices. J Clin Psychiatry. 2021;82(1):20f13804. doi: 10.4088/JCP.20f13804 33999541

[6] Hartgerink CHJ, Van Zelst M. “As-You-Go” Instead of “After-the-Fact”: a network approach to scholarly communication and evaluation. Publications. 2018;6(2):21. doi: 10.3390/publications6020021

[7] Klebel T, Reichmann S, Polka J, McDowell G, Penfold N, Hindle S, et al. Peer review and preprint policies are unclear at most major journals. PLoS One. 2020;15(10):e0239518. doi: 10.1371/journal.pone.0239518 33085678 PMC7577440

[8] Grant S, Corker KS, Mellor DT, Stewart SLK, Cashin AG, Lagisz M, et al. TOP 2025: an update to the transparency and openness promotion guidelines. Center for Open Science. 2025. doi: 10.31222/osf.io/nmfs6_v2 PMC13439764 42557573

[9] International Committee of Medical Journal Editors (ICMJE). Recommendations for the conduct, reporting, editing, and publication of scholarly work in medical journals; 2025. Available from: https://www.icmje.org/icmje-recommendations.pdf 10.12771/emj.2024.e48 PMC12093628 40704003

[10] Heidt A. AI for research: the ultimate guide to choosing the right tool. Nature. 2025;640(8058):555–7. doi: 10.1038/d41586-025-01069-0 40195514

[11] Hoch R, Clarke J. A scientific future shared with AI. PLoS Biol. 2025;23(6):e3003274. doi: 10.1371/journal.pbio.3003274 40587498 PMC12208432

